# Patient Self-Scanning for Lung Ultrasound: A Prospective Observational Study on the Feasibility and Diagnostic Accuracy of a Telemedicine Protocol

**DOI:** 10.7759/cureus.85203

**Published:** 2025-06-01

**Authors:** Katharine T Clark, Kathleen McFadden, Benjamin A Krauss, Lachlan Driver, Irene W Ma, Rachel Vivian, Jamie Gullikson, Lauren Selame, Calvin K Huang, Andrew S Liteplo, Hamid Shokoohi

**Affiliations:** 1 Emergency Medicine, Harvard Medical School, Boston, USA; 2 Internal Medicine, Massachusetts General Hospital, Harvard Medical School, Boston, USA; 3 Medicine, Stony Brook University, Renaissance School of Medicine, Stony Brook, USA; 4 Emergency Medicine, Massachusetts General Hospital, Harvard Medical School, Boston, USA; 5 Internal Medicine, University of Calgary, Calgary, CAN; 6 Emergency Medicine, Brigham and Women's Hospital, Boston, USA

**Keywords:** lung ultrasound (lus), patient-performed lung ultrasound (p-plus), remote patient monitoring, self-performed ultrasound, telemedicine, tele-ultrasound

## Abstract

Objective: To evaluate the feasibility and diagnostic accuracy of patient-performed lung ultrasound (P-PLUS) for telemedicine purposes.

Methods: This prospective observation study included patients over 18 years old who presented to a tertiary care hospital's emergency department. Patients were provided a five-minute instructional video on a US protocol of four lung zones and then performed the protocol while being monitored by a study investigator. The physician sonographer subsequently repeated the protocol. Two emergency physicians with US fellowship training blindly reviewed and independently rated image quality on a scale of one to five, with a score of three or more considered interpretable. Inter-rater reliability was estimated using the intraclass correlation coefficient. Wilcoxon-Mann-Whitney tests and chi-square tests were used to compare group differences.

Results: A total of 56 patients (45% female) were enrolled, and 417 clips were analyzed. Ten (18%) participants worked in the medical field, and 44 (79%) had at least some college education. Forty (71%) regularly used technology at work, 52 (93%) had internet access at home, and the same number had access to smartphones. Patients reported high comfort in performing self-LUS (median score: 4, interquartile range (IQR): 3.5-5) and high willingness to perform US acquisition again in the future (median score: 4, IQR: 4-5). The proportion of interpretable images was similar between the two groups except for the left hemidiaphragm (90% of provider-obtained images were interpretable vs. 45% of patient-obtained images, P = 0.002). The majority of patient-obtained images were scored between three and four and classified as interpretable. Mean image scores were significantly higher for provider-obtained images (P < 0.05). Inter-rater reliability between the two raters was good (intraclass correlation coefficient (ICC)= 0.80, 95% CI 0.76-0.84).

Conclusion: Patients can independently obtain interpretable LUS images in all views with minimal video-based instruction. The ability of patients to obtain interpretable LUS images with minimal tele-guidance, as well as their high levels of comfort and willingness to perform the procedure, support the potential use of P-PLUS in home-based and remote patient care.

## Introduction

Lung ultrasound (LUS) is a valuable tool for evaluating patients with dyspnea in the emergency department [1-3], as it has a higher sensitivity than chest X-ray in detecting various pulmonary conditions [3-7] and is cost-effective, portable, and time-efficient. During the recent COVID-19 pandemic, LUS was particularly useful in evaluating patients with suspected COVID-19 infection and helped to reduce provider exposure to infectious patients [8, 9-13].

Recent studies have demonstrated that novice patients can be taught to self-perform LUS with the guidance of experts via telemedicine [14-18], showing the feasibility of self-performed US surveillance of lung health in at-risk patients. However, these studies have required the real-time direction and supervision of expert ultrasonographers, and it is not yet known if patients can self-perform LUS remotely without the aid of real-time mentoring by experts. The ability of patients to self-perform LUS without real-time expert guidance could decrease both the monetary costs and opportunity costs (in terms of time and provider availability) associated with retaining a clinician on call to guide scanning.

This study aims to evaluate the feasibility and diagnostic accuracy of patient-performed LUS (P-PLUS) using minimal video-based instruction, without real-time expert guidance. We hypothesized that patients could independently acquire diagnostically useful LUS images with minimal tele-guidance and that these images would be accurately interpreted by expert sonographers. By assessing image quality, patient comfort, and usability across different lung zones, this study explores whether P-PLUS could support remote patient monitoring, particularly for individuals with respiratory illnesses who require ongoing evaluation. Our findings may inform the development of patient-centered telemedicine protocols and expand access to point-of-care imaging in home and low-resource settings, offering a potential shift in how US is used outside traditional clinical environments.

## Materials and methods

Participant enrollment

This study was a prospective, observational study enrolling English-speaking patients aged 18 or older presenting to the ED of a tertiary care center. In a convenience-based sampling method, subjects, some with pulmonary symptoms and others without, were identified by examining the chief complaints in the ED electronic medical record and after discussion with the clinical team caring for enrolled patients. Patients who were hemodynamically unstable, had altered mental status, or had pulmonary symptoms secondary to trauma were excluded. Patients were not excluded on the grounds of race, gender, or ethnicity. Informed consent for participation was obtained for all participants by a research team member. The Institutional Review Board (IRB) of Mass General Brigham, Boston, MA, approved this study (approval number: 2020P002611). Because this was a prospective feasibility study aimed at assessing whether patients could independently perform LUS using video instruction, a formal sample size calculation was not performed, as the goal was to observe technical capability and general trends in image quality.

Patients watched a five-minute instructional video via a portable handheld iPad (Apple Inc., Cupertino, CA). Participants could pause, rewind, and replay the video as needed. The video described performing a four-zone LUS scan (right superior anterior, left superior anterior, right hemidiaphragm, left hemidiaphragm) to obtain a clip of the anterior chest in the midclavicular line and the lateral chest in the midaxillary line at the anticipated level of the lung-diaphragm interface. In our protocol, the left anterior chest refers to scanning the upper and mid portions of the thorax in the midclavicular line, while the left hemidiaphragm refers more specifically to the lower chest zone where the lung and diaphragm interface, typically approached from a more inferior and lateral angle around the posterior axillary line. Participants were provided with a handout to illustrate the technique. The handout included images that patients were expected to obtain to be used as a reference, as desired. The ultrasound machines used were standard equipment in the ED, and we dedicated a Mindray TE7 (Mindray North America, Mahwah, NJ) for the purpose of completing this study. The Mindray TE7 ultrasound system was used with a Mindray C62 convex transducer (Mindray North America). Scans were performed using the standard lung preset.

Scanning protocol

The scanning protocol for the study included the following steps: First, patients watched an instructional video on a four-zone LUS protocol. Using a Mindray C62 ultrasound transducer (Mindray North America), patients obtained images of the four zones of the lung, recording short clips when they felt they had achieved an adequate image at each point. The six-second clips were labeled with zones 1-4, corresponding to the specific zone being scanned, and the images were wirelessly stored on the Visage Imaging System (Visage Imaging, Inc., San Diego, CA). Next, an expert sonographer repeated the four-point LUS protocol, using the same ultrasound transducer and following the same instructions as the patients. Finally, the findings noted by the investigator were shared with the clinical team in real time.

Data collection and scoring

After the images were obtained, patients completed a survey using a five-point Likert scale, which examined their willingness and comfort to perform the study independently. They were also asked to rate their hypothetical willingness to complete self-scans as part of home monitoring. Demographic data and clinical information were collected from the patient's electronic medical records, including their levels of education and previous experience with technology.

The images were then deidentified, and two fellowship-trained attending physicians who were blind to whether the images were obtained by the patient or the sonographer independently reviewed and scored the images. The physicians rated the image quality on a Likert scale from one to five, with a score of three or higher considered interpretable. They evaluated the images for diagnostic quality and recorded any pathological findings, such as pleural effusion, B lines, subpleural consolidation, or pleural irregularity.

Statistical analyses

Survey questions used a five-point Likert scale to assess comfort and willingness, though the instrument was not formally validated. Statistical analyses were performed using Wilcoxon-Mann-Whitney and chi-squared tests to compare group differences in the evaluation of interpretable videos and quality scores of patient-acquired scans versus physician sonographer scans. Inter-rater reliability between the two reviewers was also assessed using the intraclass correlation coefficient (ICC). More specifically, Wilcoxon-Mann-Whitney tests were used to compare image quality scores due to the ordinal nature of the Likert scale ratings and non-normal distribution of quality scores. Chi-square tests were used to compare the proportions of interpretable images. These non-parametric tests were selected to accommodate the categorical and non-normally distributed data. All analyses were conducted using SAS v9.4 (SAS Institute Inc., Cary, NC) and IBM SPSS Statistics software, version 26 (IBM Corp., Armonk, NY).

## Results

Fifty-six patients participated in this prospective observational study (Table 1).

**Table 1:** Baseline characteristics of the patients who participated in the study Presented as N (%) unless otherwise stated; *Participants identifying as Non-Hispanic also self-identified as one of the following races, including Non-Hispanic White, Non-Hispanic Black, Non-Hispanic Asian, Non-Hispanic American Indian or Alaska Native, or Non-Hispanic Native Hawaiian or Other Pacific Islander. IQR: interquartlie range

Baseline characteristics	N (%)
Mean age (SD)	54.2 (17.0)
Sex	
Male	31 (55)
Female	25 (45)
Mean body mass index (SD)	28.3 (5.2)
Ethnicity	
Hispanic	5 (9)
Non-Hispanic*	49 (91)
Self-identified race	
White	46 (84)
Black or African American	5 (9)
Other	4 (7)
Self-reported English proficiency	
Native speaker	51 (91)
Non-native speaker but fluent in English	5 (9)
Preferred language	
English	55 (98)
Spanish	1 (2)
Other	0
Highest level of education	
High school or General Educational Development (GED) diploma	12 (21)
Some college	14 (25)
College degree	16 (29)
Postgraduate degree	14 (25)
Works in the medical field	10 (18)
Has a family member who works in the medical field	24 (43)
Use technology heavily at work (computers, cell phones, tablets)	40 (71)
Median comfort using computer (1-5)	4.5 (IQR 3-5)
Has had a lung or cardiac ultrasound (echo)	30 (54)
Handedness	
Right	51 (91)
Left	4 (7)
Plays video games	11 (20)
Sends pictures or videos via phone or computer	37 (66)
Has access to a smartphone	37 (93)
Has high-speed internet at home	52 (93)

A total of 417 LUS scan clips were collected from patient-performed and physician-sonographer-acquired scans. For the novice scanners, the median duration of the scans was seven minutes (interquartile range (IQR): four minutes to 10.5 minutes), with a range of three to 19 minutes. The majority of participants were able to find the correct probe, and all participants were able to apply the gel for the US. Fifty-three (95%) of the participants could press the US machine's record button. Most participants watched the instructional video only once (IQR: 1-1; range: 1-2) (Table 2).

**Table 2:** Scanning ability of the participants IQR: interquartile range; LUS: lung ultrasound

Patients' scanning characteristics			
Median scan duration (N minutes, IQR, range)	7	4-10.5	3-19
Number able to find the correct probe (N (%))	52 (93)		
Number able to apply gel (N (%))	56 (100)		
Number able to press record (N (%))	53 (95)		
Number of times video viewed (N, IQR, range)	1	1-1	1-2
Median comfort with performing LUS (N, IQR, range)	4	3.5-5	1-5
Median willingness to perform LUS (N, IQR, range	4	4-5	1-5

In reference to the potential future home-based individual scans, 35 (63%) felt that the video was sufficient instruction alone, and 18 (33%) expressed they may need some access to online material or virtual expert instruction if they had to repeat scans at their own home (Table 3).

**Table 3:** Anticipated type of assistance needed to perform LUS in the future LUS: lung ultrasound

Type of assistance needed	Video alone	Online materials	Virtual expert	In-person expert	Complete Inability to perform
N(%)	35 (63)	11 (20)	7 (13)	3 (5)	0 (0)

The majority were also able to complete the four-point lung US protocol using the instructional video alone, with the exception of the left lung base, where 12 (21%) required additional verbal instructions and one (2%) required manual assistance. Overall, patients reported high comfort in performing the self-LUS (median score: 4, IQR: 3.5-5) and high willingness to perform it again in the future (median score: 4, IQR: 4-5) (Table 4).

**Table 4:** Type of instruction required to obtain each view of LUS Presented as N (%) unless otherwise stated; LUS: lung ultrasound

Location of image acquisition	Right anterior chest	Right lateral chest	Left anterior chest	Left lateral chest
Number of complete scans	54 (96)	39 (70)	52 (93)	35 (63)
Type of instruction required				
Video alone	35 (63)	41 (75)	44 (79)	42 (75)
Minimum instruction	16 (29)	9 (16)	8 (14)	8 (14)
Multiple instructions	4 (7)	4 (7)	3 (5)	4 (7)
Manually assist the patient	1 (2)	4 (7)	0	1 (2)
Unable to perform LUS	0 (0)	1 (2)	1 (2)	1 (2)

In general, the quality of cine loops obtained by the experts was higher than that obtained by the patients. Differences in quality reached statistical significance for all areas of the chest wall. The left hemidiaphragm had the highest proportion of interpretable patient-obtained images, while the right anterior chest had the lowest. The lower interpretability rates for patient-acquired images of the left hemidiaphragm likely reflect the increased technical difficulty in locating this interface, especially for novice users without real-time guidance. Yet, despite differences in quality, the majority of patient-performed clips were still deemed interpretable. The percentage of interpretable images was higher for expert-obtained scans (Table 5).

**Table 5:** Median quality score and percentage of interpretable lung ultrasound cine loops performed by patients compared with sonographers Quality is presented as the median quality score (out of five) and interquartile range (IQR). Wilcoxon-Mann-Whitney (MW) and chi-squared tests were used to compare group differences in the evaluation of interpretable videos and quality scores of patient-acquired scans versus physician sonographer scans. avg: average; R: right; L: left

Chest location	Physician-performed	Patient-performed	Wilcoxon MW p-value
Median R anterior chest avg quality score	4.5 IQR 3.5-5.0	3.5 IQR 2.5-4.0	<0.0001
Median R lateral chest avg quality score	3.5 (3.0-4.5)	3.0 (2.5-4.0)	0.0023
Median L anterior chest avg quality score	4.0 (4.0-4.5)	3.5 (3.0-4.0)	<0.0001
Median L lateral chest avg quality score	3.5 (2.5-4.0)	2.5 (1.5-3.5)	0.0004
Using an average quality score of 3 or more as interpretable quality (Chi-square p-values presented)			
R anterior chest	50/55 (91%)	39/56 (60%)	0.64
R lateral chest	46/55 (84%)	32/56 (57%)	0.96
L anterior chest	53/56 (95%)	45/56 (80%)	0.38
L lateral chest	39/54 (72%)	22/55 (40%)	0.58

Inter-rater reliability of image quality scores was good, with an ICC of 0.80 (95% CI: 0.76-0.84; p<0.001). Agreement between the two raters regarding interpretability varied from moderate to good, depending on the region, with an overall agreement for the decision of interpretability being moderate (Kappa = 0.54, 95% CI 0.44-0.63, p<0.001) (Table 6).

**Table 6:** ICC with agreement by area of chest wall. Reviewer agreement of scores is good when all clips were combined:  ICC = 0.80, 95% CI 0.76-0.84; P<0.001. Overall agreement for decision regarding interpretable or not (3 and above or <3); considered only Moderate:  Kappa = 0.54, 95% CI 0.44-0.63; P<0.001 ICC: intraclass correlation coefficients; MD: Doctor of Medicine; R: right; L: left

Scan location	ICC	95%% CI	p-value	ICC interpretation
R anterior chest by the patient	0.84	0.73-0.91	<0.001	Good
R anterior chest by the MD	0.60	0.33-0.77	<0.001	Moderate
R lateral chest by the patient	0.85	0.74-0.91	<0.001	Good
R lateral chest by the MD	0.60	0.32-0.77	<0.001	Moderate
L anterior chest by the patient	0.65	0.41-0.80	<0.001	Moderate
L anterior chest by the MD	0.56	0.26-0.74	0.001	Moderate
L lateral chest by the patient	0.86	0.75-0.92	<0.001	Good
L lateral chest by the MD	0.59	0.29-0.76	<0.001	Moderate

## Discussion

In this study, we evaluated the feasibility and diagnostic accuracy of P-PLUS for telemedicine purposes. The results of this study demonstrate that with minimal video-based instruction, patients can obtain interpretable LUS images in all views. This is a promising finding, as it suggests that P-PLUS has the potential to be used as a tool for remote patient monitoring in the future. This includes monitoring clinically stable patients from home using handheld US devices. While interest in remote imaging was accelerated by the COVID-19 pandemic, the value of P-PLUS extends to other chronic respiratory and cardiovascular conditions. In particular, patients with heart failure or chronic obstructive pulmonary disease (COPD) may benefit from routine, at-home scanning as part of a remote care model. This is particularly important for patients with chronic pulmonary infections and stable pulmonary edema whose clinical course may be prolonged and have unpredictable changes in acuity [18, 19].

However, the results of this study also showed that the proportion of interpretable images was lower for patient-obtained images compared to expert-obtained images. This finding is consistent with previous studies showing that novice ultrasonographers may have difficulty obtaining high-quality images [17-19]. Additionally, the mean image scores were significantly higher for expert-obtained images, indicating a potential difference in image quality between patient-obtained and expert-obtained images. However, previous studies found that in almost all cases, novice users who received guidance from experts via live tele-US were able to independently obtain interpretable images of the lateral and anterior lung chest [15, 17]. In contrast, our study involved patients independently obtaining images without live or remote guidance. This suggests that patient-performed US may be a feasible and efficient alternative for home-based care and remotely performed US. Our study highlights a more scalable approach that could reduce reliance on clinician supervision in future tele-US applications. Given variability in patient performance, this points to the need for targeted training tools or supplemental guidance to ensure diagnostic reliability. Future studies should also compare patient performance with and without tele-US support to determine whether real-time feedback improves consistency and quality.

The results of the study may be limited by its single-center design and small sample size, which may not be representative of the larger population and may not have sufficient power to detect significant differences. This study was designed as a preliminary investigation of feasibility and was not powered to detect outcome differences. Therefore, a formal sample size calculation was not performed. Future multi-site studies with larger and more diverse populations are needed to validate these findings and assess performance across varying clinical and demographic contexts. The lack of diversity in the study sample, for example, which was predominantly white, English-speaking, and highly educated, may limit the generalizability of the results to other demographic groups, including more diverse or underserved populations. Future research should aim to include a broader demographic range to assess usability and performance across different patient backgrounds. Participants may also have been more motivated or technologically comfortable than the general population, introducing a possible selection bias that further limits generalizability. Furthermore, although patient diagnosis was not formally collected or analyzed in this study, future research should evaluate whether specific clinical conditions, such as pulmonary disease, obesity, or prior imaging experience, affect image quality or patient performance. Factors such as age, prior technology use, and clinical stability may have impacted performance but were not adjusted for in this analysis. Future work should explore these confounding factors through stratified analysis or multivariate modeling. 

Self-reported measures of diagnostic accuracy may be subject to bias and may not be as reliable as those obtained by a trained professional. The study may not have adequately controlled for other factors that could influence the accuracy of the diagnostic test, such as the patient's knowledge or experience with US, physical condition, or other medical conditions. The study has not adequately evaluated the long-term effectiveness of P-PLUS in improving diagnostic accuracy and patient outcomes.

Additionally, for image acquisition, the Mindray TE7 was selected primarily because it was the standard US machine readily available in our ED at the time of the study. Although the TE7 is not a handheld device, it allowed us to evaluate patient scanning in a consistent and controlled clinical environment, serving as a practical first step for assessing feasibility. Because the TE7 is a cart-based US system, it may not reflect the equipment available in home or low-resource environments. Future studies should incorporate handheld devices to better assess real-world applicability in remote care settings, as well as to better reflect real-world home use and assess whether device type affects image quality, usability, or patient performance.

Although expert sonographers performed scans using the same protocol, these were not intended to serve as a formal control group. Future studies should include provider-performed scans under similar conditions as a benchmark for image quality and diagnostic utility. While this study focused on technical feasibility, future research should explore the downstream clinical value of P-PLUS, including its effect on clinical decision-making, time to diagnosis, and patient outcomes in home-monitoring scenarios.

Despite these limitations, the high levels of comfort and willingness to perform P-PLUS among patients suggest that this method of remote patient monitoring, which could be used for patients with chronic respiratory conditions or those recovering from infections such as COVID-19, has the potential to be well-received by patients. While this study supports the feasibility of P-PLUS, further research is needed to determine whether such scans can influence clinical decision-making or detect early changes in disease progression and whether that improves patient outcomes in chronic care settings and in real-world clinical settings. In addition, the use of artificial intelligence (AI) in interpreting self-acquired LUS images has the potential to improve the accuracy and efficiency of P-PLUS in remote patient care [20]. Further research is needed to evaluate AI use in P-PLUS and determine the optimal training and instruction methods for patients to perform P-PLUS with the highest accuracy. Future studies should evaluate how AI-guided feedback or automated interpretation might support novice users and improve diagnostic reliability in self-performed LUS.

## Conclusions

In conclusion, our findings show that patients can successfully obtain interpretable LUS images in all views with minimal video-based instruction. While some difficulty was observed in obtaining images of the left hemidiaphragm, overall, patients demonstrated a high level of comfort and willingness to perform the procedure. These results support the potential use of P-PLUS as a tool for remote patient care. Further research is needed to optimize the training and instruction methods for patients to perform P-PLUS with the highest accuracy and to evaluate the use of AI in interpreting self-acquired LUS images.
